# Self-perceived psychological stress and ischemic stroke: a case-control study

**DOI:** 10.1186/1741-7015-7-53

**Published:** 2009-10-01

**Authors:** Katarina Jood, Petra Redfors, Annika Rosengren, Christian Blomstrand, Christina Jern

**Affiliations:** 1 Institute of Neuroscience and Physiology, the Sahlgrenska Academy at University of Göteborg, Göteborg, Sweden; 2 Department of Medicine, Sahlgrenska University Hospital/Östra, Göteborg, Sweden; 3 Department of Clinical Genetics, Sahlgrenska University Hospital, Göteborg, Sweden

## Abstract

**Background:**

A growing body of evidence suggests that psychological stress contributes to coronary artery disease. However, associations between stress and stroke are less clear. In this study, we investigated the possible association between ischemic stroke and self-perceived psychological stress, as measured by a single-item questionnaire, previously reported to be associated with myocardial infarction.

**Methods:**

In the Sahlgrenska Academy Study on Ischemic Stroke (SAHLSIS), 600 consecutive patients with acute ischemic stroke (aged 18 to 69 years) and 600 age-matched and sex-matched population controls were recruited. Ischemic stroke subtype was determined according to Trial of Org 10172 in Acute Stroke Treatment (TOAST) criteria. Self-perceived psychological stress preceding stroke was assessed retrospectively using a single-item questionnaire.

**Results:**

Permanent self-perceived psychological stress during the last year or longer was independently associated with overall ischemic stroke (multivariate adjusted odds ratio (OR) 3.49, 95% confidence interval (CI) 2.06 to 5.93). Analyses by stroke subtype showed that this association was present for large vessel disease (OR 3.91, 95% CI 1.58 to 9.67), small vessel disease (OR 3.20, 95% CI 1.64 to 6.24), and cryptogenic stroke (OR 4.03, 95% CI 2.34 to 6.95), but not for cardioembolic stroke (OR 1.48, 95% CI 0.64 to 3.39).

**Conclusion:**

In this case-control study, we found an independent association between self-perceived psychological stress and ischemic stroke. A novel finding was that this association differed by ischemic stroke subtype. Our results emphasize the need for further prospective studies addressing the potential role for psychological stress as a risk factor for ischemic stroke. In such studies ischemic stroke subtypes should be taken into consideration.

## Background

A growing body of evidence suggests that psychosocial factors including psychological stress contribute to cardiovascular disease [1]. With respect to coronary artery disease, association with several constructs representing various concepts of psychological stress have been reported from epidemiological studies [2-8]. In the INTERHEART study [6], a large case-control study with participants from 52 countries, the subjective perception of psychological stress was measured using a semiquantitative single item question. Interestingly, this simple measure showed a strong correlation to other constructs measuring various aspects of stress, including financial stress, stressful life events, and locus of control [6]. Moreover, an independent association to myocardial infarction was found that was consistent across regions and ethnic groups [6].

Although psychological stress is commonly perceived as a risk factor for stroke among the lay public [9,10], scientific data on stress as a risk factor for stroke are limited. A few prospective and case-control studies have reported severe self-perceived psychological stress, various stressful life events, and failure to find successful strategies in stressful situations to be independently associated with an increased risk of stroke [11-16]. Other studies measuring perceived psychological stress, psychological distress, or recent stressful life events did not detect any significant association with non-fatal or total stroke [17-20]. However, in three of these latter studies, subgroup analyses revealed a significant association with fatal stroke [18-20], suggesting an association confined to severe cases or non-survival. An association between severe self-perceived stress and stroke mortality has also been reported from a large Japanese study [4]. In this study the association was confined to women, whereas another study found an association only in men [20].

Because there is considerable pathological heterogeneity in stroke, different endpoints under study (for example, stroke mortality, total stroke, non-hemorrhagic stroke) may contribute to the heterogeneous findings. Many studies did not differentiate between ischemic and hemorrhagic stroke [12,13,19,20], and no study characterized ischemic stroke subtypes. However, differences between ischemic stroke subtypes have been reported from a small case-control study measuring type A behavior [21]. In this study, a high tenseness score was associated with ischemic stroke due to large vessel disease (LVD), but not with ischemic stroke due to small vessel disease (SVD).

Using data collected in the Sahlgrenska Academy Study on Ischemic Stroke (SAHLSIS) [22], a case-control study including ischemic stroke patients with careful phenotyping pertaining to stroke subtypes, we investigated self-perceived psychological as measured by the single-item previously reported to be associated with myocardial infarction in INTERHEART [6], in relation to ischemic stroke, the four major stroke subtypes, and functional outcome after 3 months.

## Methods

### Study population

The study population includes the participants in the SAHLSIS cohort, which has been described elsewhere [22]. In short, Caucasian patients presenting with acute ischemic stroke before the age of 70 years were consecutively recruited from four stroke units in Western Sweden. Patients were recruited regardless of previous cardiovascular or cerebrovascular disease. Of 645 eligible stroke patients, 29 were unwilling to participate and 16 died before the patient or a next of kin could give detailed informed consent.

For each case, one Caucasian control without clinical atherothrombotic disease, matched for age (± 1 year), sex, and geographical residence area was randomly selected from the population. If a selected control did not respond, refused to participate or was ineligible because of prior cardiovascular disease, a second and then a third matched control was invited. Of the 1,107 selected controls, 208 did not respond, 191 were unwilling to participate, and 108 were excluded because of a history of stroke, coronary or peripheral artery disease, or signs of ischemic heart disease on resting electrocardiogram (ECG) according to the Minnesota code (1982).

The study was approved by the Ethics Committee of Göteborg University, Sweden. All participants gave their written informed consent. Next of kin consented for those participants who were unable to communicate.

### Stroke subtyping

All patients were examined by a physician trained in stroke medicine, both during the acute stage and at a follow-up visit after 3 months. All patients underwent an ECG and neuroimaging with computed tomography (CT) and/or magnetic resonance imaging (MRI) during the acute stage. Additional diagnostic analysis was performed when clinically indicated as described previously [22]. Neurological deficits were assessed according to the Scandinavian Stroke Scale. Patients included at Sahlgrenska University Hospital from March 1999 to December 2003 (n = 328) were assessed with respect to visuospatial neglect as previously described [23,24]. Stroke subtype was assessed according to modified Trial of Org 10172 in Acute Stroke Treatment (TOAST) criteria [22,25]. The analysis by subtypes was confined to the four major subtypes: LVD, SVD, cardioembolic (CE), and cryptogenic stroke. Cryptogenic stroke was defined when no cause was identified despite an extensive evaluation. Stroke outcome was evaluated after 3 months using the modified Rankin scale (mRS, score 0 to 6). The score was dichotomized for death or dependency (score of 3 to 6) versus a favorable outcome (score of 0 to 2).

### Data collection and risk factor definition

Self-perceived psychological stress during the previous years was assessed with a single-item questionnaire that has been used in epidemiological studies in Göteborg, Sweden since 1970 [2,11] and in the INTERHEART study [6]. In this questionnaire stress is described as feeling tense, irritable, anxious, or as having sleeping difficulties as a result of conditions at home or at work. Participants were asked to report how often they had felt stress, using the following incremental or graded response options: (1) never, (2) at some period, (3) at some period during the last 5 years, (4) at several periods during the last 5 years, (5) permanent stress during the last year, or (6) permanent stress during the last 5 years. Patients responded to the question 1 to 10 days after the acute event and controls at the time of their examination. Permanent, but not periodic, stress as assessed with this measure, has previously been found to predict myocardial infarction and overall stroke in prospective studies [2,11]. Accordingly, in the present multivariable regression analyses and in subtype analysis, this variable was dichotomized so that subjects reporting permanent self-perceived psychological stress for ≥1 year formed one category (score 5 to 6) and all the others formed the reference category (score 1 to 4). Information on self-perceived psychological stress was missing in 7 controls and 34 cases. Among these 34 cases, 3 could not complete the questionnaire due to intervening death, 10 due to unconsciousness/disorientation or severe aphasia, and 21 due to unwillingness to answer the question. Thus, 566 cases and 593 controls were included in the present analysis.

Information on other vascular risk factors was collected and defined as previously described [22]. In short, examinations were performed during the acute stage and at a follow-up visit after 3 months. Controls were examined once. Hypertension was defined as pharmacological treatment for hypertension, and/or systolic blood pressure ≥160 mm Hg, and/or diastolic blood pressure ≥90 mm Hg. Diabetes was defined by diet or pharmacological treatment, fasting plasma glucose ≥7.0 mmol/l, or fasting blood glucose ≥6.1 mmol/l. Hyperlipidemia was defined by pharmacological treatment, total fasting serum cholesterol >5.0 mmol/l, and/or low-density lipoprotein (LDL) >3.0 mmol/l. Waist and hip circumferences were measured using flexible measuring tape at the level of the umbilicus, and at the level of the bilateral greater trochanters, respectively. Waist to hip ratio (WHR) was defined as waist divided by hip circumference. Among cases, anthropometric measurements performed during the acute phase were used for calculation of WHR, whereas 3-month follow-up measures were used to define hypertension, diabetes and hyperlipidemia. Smoking history was coded as current versus never or former (smoking cessation at least 1 year before inclusion in the study). Leisure time physical activity before stroke was dichotomized as sedentary versus regular, moderate physical activity of at least 4 h/week. A positive family history of stroke was defined as history of stroke in a first-degree relative. Socioeconomic status was classified according to the occupation-based Swedish socioeconomic classification system. The score was dichotomized with employed and self-employed professionals, higher civil servants, executives, and intermediate non-manual employees forming one category (corresponding to higher education) and assistant non-manual employees and manual workers forming a second category (corresponding to lower education).

### Statistical methods

Descriptive statistics are presented as frequencies or mean values and standard deviations (SD). Differences between groups were examined with the χ^2 ^test for proportions and with Student t test for continuous variables. Multivariate odds ratios (ORs) and 95% confidence intervals (CIs) for self-perceived psychological stress were calculated using conditional logistic regression analysis. The multivariable model included age, hypertension, smoking status, diabetes, hyperlipidemia, WHR, family history of stroke, self-perceived psychological stress, leisure time physical activity, and occupational class. ORs were calculated for overall ischemic stroke and for the four major subtypes. Each subtype was compared with the whole control group, and unconditional logistic regression analysis was therefore applied for calculation of ORs among subtypes. ORs for death or dependency (defined as mRS 3 to 6) 3 months after stroke were calculated in cases, using binary logistic regression. This model included age, sex, hypertension, smoking status, diabetes, hyperlipidemia, WHR, family history of stroke, self-perceived psychological stress, leisure time physical activity, occupational class, and stroke subtype. Data were analyzed using SPSS V. 12.0 (SPSS, Chicago, IL, USA). Results were considered significant at *P *< 0.05 using two-tailed tests.

### Missing values

Information about diabetes was missing in 2 participants, hypertension in 7, hyperlipidemia in 46, WHR in 45, leisure time physical activity in 33, family history of stroke in 31, and occupational class in 31. In 29 patients information about 3-month outcome was missing. In the multivariable logistic regression, missing values for continuous variables were replaced by mean values, and for categorized variables observations with missing values were excluded.

## Results

The mean age of cases and controls was 56.3 (18 to 69) years and 64% were males. The distribution of stroke subtypes was as follows: LVD (n = 70, 12%); SVD (n = 120, 21%); CE (n = 90, 16%); cryptogenic stroke (n = 154, 27%); other determined etiology (n = 48, 8%); and undetermined stroke (n = 84, 15%). Crude ORs of overall ischemic stroke for the different categories of self-perceived psychological stress are shown in Table 1. No association between ischemic stroke and periods of self-reported stress was detected. In contrast, permanent stress during the last year or longer showed significant association to an increased risk of ischemic stroke.

**Table 1:** Crude odds ratios (95% confidence interval (CI)) of ischemic stroke for self-perceived psychological stress

	**Cases/controls**	**Odds ratio (95% CI)**
Never experienced stress	56/51	1.00 (reference)
Some period of stress	136/187	0.66 (0.43 to 1.03)
Some period of stress during the last 5 years	89/126	0.64 (0.40 to 1.03)
Several periods of stress during the last 5 years	159/183	0.79 (0.51 to 1.22)
Permanent stress during the last year	46/17	2.46 (1.26 to 4.83)**
Permanent stress during the last 5 years	80/29	2.51 (1.42 to 4.44)**

**P < 0.01.

Risk factor profiles according to the dichotomized categories of self-perceived psychological stress are given in Table 2. Subjects reporting permanent psychological stress were younger, more often female, more frequently smokers, and were also less active during leisure time as compared with subjects reporting no or periods of psychological stress. After adjustment for age and gender in a binary logistic regression model, significant associations between permanent self-perceived psychological stress and hypertension (*P *< 0.01), diabetes (*P *< 0.05), WHR (*P *< 0.01), and low physical activity (*P *< 0.001) were found. Smoking, hyperlipidemia, occupational class, and family history of stroke did not show any significant association to permanent self-perceived stress after adjustment (data not shown).

**Table 2:** Risk factors by categories of self-perceived psychological stress

	**Self-perceived psychological stress**		***P *value**
		
	**Never/periods, n = 987**	**Permanent ≥1 year, n = 172**	
Mean age, years (SD)	57 (10)	53 (11)	<0.001
Male sex, n (%)	652 (66)	80 (46)	<0.01
Hypertension, n (%)	466 (46)	90 (53)	0.22
Smokers, n (%)	265 (27)	60 (35)	<0.05
Diabetes, n (%)	113 (11)	28 (16)	0.08
Hyperlipidemia, n (%)	679 (71)	119 (72)	0.91
Occupation, lower education, n (%)	558 (58)	88 (53)	0.27
Sedentary leisure time, n (%)	104 (11)	40 (24)	<0.001
WHR, mean (SD)	0.932 (0.074)	0.934 (0.070)	0.77
Family history of stroke, n (%)	317 (33)	60 (37)	0.35

Differences between groups were examined with the χ^2 ^test for proportions and with the Student t test for continuous variables.

SD = standard deviation; WHR = waist to hip ratio.

The results of the conditional univariate and multivariate logistic regression analyses with ischemic stroke as outcome variable are given in Table 3. Permanent self-perceived stress during the last year or longer showed an independent association to an increased risk of overall ischemic stroke. Similar associations were seen in men and women (adjusted OR 3.76, 95% CI 1.58 to 8.93 and 3.28, 95% CI 1.65 to 6.52 for women and men, respectively), and in participants under or above the median age of 58.8 years (OR 3.71, 95% CI 1.76 to 7.81 and OR 3.89, 95% CI 1.70 to 8.86, for under and above the median age, respectively).

**Table 3:** Odds ratios (ORs) and 95% confidence intervals (CIs) of permanent self-perceived psychological stress and vascular risk factors for ischemic stroke

	**Univariate**	**Multivariate**^**a**^
Permanent self-perceived psychological stress^b^	3.38 (2.31 to 4.96)***	3.49 (2.06 to 5.93)***
Hypertension	2.75 (2.09 to 3.60)***	2.21 (1.53 to 3.20)***
Smoking	3.02 (2.23 to 4.08)***	3.04 (1.98 to 4.67)***
Diabetes	3.78 (2.47 to 5.77)***	2.61 (1.51 to 4.52)***
Hyperlipidemia	1.64 (1.22 to 2.20)**	1.77 (1.19 to 2.63)**
Occupation, lower education	1.51 (1.18 to 1.93)**	1.28 (0.92 to 1.80)
Regular leisure time physical activity^c^	0.34 (0.23 to 0.51)***	0.66 (0.39 to 1.13)
WHR, per 1 SD increase	1.71 (1.45 to 2.02)***	1.36 (1.09 to 1.70)**
Family history of stroke	1.77 (1.35 to 2.32)***	1.69 (1.16 to 2.43)**

**P < 0.01; ***P < 0.001; ^a^conditional logistic regression with age, self-perceived psychological stress, hypertension, smoking status, diabetes, hyperlipidemia, occupation, leisure time physical activity, WHR, and family history of stroke included in the model; ^b^permanent self-perceived stress during the last year or longer; ^c^regular, moderate physical activity of at least 4 h/week.

SD = standard deviation; WHR = waist to hip ratio.

Patients were consecutively recruited regardless of previous cardiovascular or cerebrovascular disease. After exclusion of patients with previous atherothrombotic disease (n = 184), the independent association between self-reported stress and ischemic stroke persisted (adjusted OR 3.50, 95% CI 2.29 to 5.34, data not shown).

Subgroup analysis showed that the association between self-perceived permanent stress and ischemic stroke was present regardless of the affected hemisphere, presence of aphasia, and visuospatial neglect. Multivariate ORs were 3.13, 95% CI 1.90 to 5.16 and 3.47, 95% CI 2.01 to 5.99 for left (n = 260) and right (n = 203) hemispheric lesions, respectively, 3.21, 95% CI 1.71 to 6.03, and 2.84, 95% CI 1.81 to 4.47 for patients with (n = 122) and without (n = 396) aphasia, respectively, and 4.80, 95% CI 2.32 to 9.99 and 3.42, 95% CI 2.13 to 5.49 for patients with (n = 82) and without (n = 246) visuospatial neglect, respectively (data not shown).

Univariate analysis by ischemic stroke subtypes showed that permanent self-perceived psychological stress during the last year or longer was associated with increased risk for all ischemic stroke subtypes. After adjustment for other vascular risk factors, the association persisted for LVD, SVD, and cryptogenic stroke, but not for CE stroke (Table 4).

**Table 4:** Odds ratios (ORs) and 95% confidence intervals (CIs) of permanent self-perceived psychological stress during the last year or longer for the major ischemic stroke subtypes

	**Stroke subtype (cases/controls)**			
	
	**LVD (70/593)**	**SVD (120/593)**	**CE (90/593)**	**Cryptogenic stroke (154/593)**
Crude	2.71 (1.38 to 5.32)**	3.13 (1.84 to 5.34)***	2.34 (1.27 to 4.47)**	4.31 (2.70 to 6.88)***
Adjusted^a^	3.91 (1.58 to 9.67)**	3.20 (1.64 to 6.24)***	1.48 (0.64 to 3.39)	4.03 (2.34 to 6.95)***

**P < 0.01; ***P < 0.001; ^a^Multivariable logistic regression analysis adjusted for age, sex, hypertension, smoking status, diabetes, hyperlipidemia, occupation, leisure time physical activity, waist to hip ratio and family history of stroke.

CE = cardioembolic stroke; LVD = large vessel disease; SVD = small vessel disease.

As previously reported by SAHLSIS, functional outcome after 3 months was dependent on stroke subtype [22]. Stroke subtype was therefore entered into the multivariable model examining the influence of permanent self-perceived psychological stress on outcome. No association between stress and outcome was observed (OR of death or dependency for permanent self-perceived psychological stress 1.37, 95% CI 0.79 to 2.37).

## Discussion

In the present study, we found an independent association between acute ischemic stroke and self-perceived psychological stress as measured by a single-item questionnaire previously reported to be associated with myocardial infarction [6]. We also found that the independent association differed by ischemic stroke subtype. In contrast, no association between self-perceived stress and functional outcome after 3 months was observed.

Our results from the present case-control study are supported by results from a prospective study of middle-aged men using the same questionnaire for assessment of self-reported psychological stress [11]. In that study, subjects, whom at baseline reported permanent stress during the last 5 years, had an increased risk of stroke after 12 years of follow-up. However, in that study it was not altogether possible to distinguish between ischemic and hemorrhagic stroke, and the strongest association was found among unspecified cases. Thus, our result supports an independent association between self-perceived stress and ischemic stroke in men. In addition, we found a similar association in women.

Ischemic stroke is a heterogeneous disease, and subgroup analysis with respect to etiological stroke subtypes was therefore performed. Interestingly, we found differences between subtypes with an independent association between permanent self-perceived stress for LVD, SVD, and cryptogenic stroke, but not for CE stroke. To the best of our knowledge, this is the first study investigating the influence of self-perceived stress in different ischemic stroke subtypes. Differences in type A behavior between stroke subtypes have been reported from a small Korean case-control study [21]. In this study, an independent association to a high tenseness score of type A behavior was only found for LVD. However, the sample size was too small to investigate CE and undetermined stroke. In contrast to the present results, no significant association was observed for SVD. Obviously, qualitative differences between type A behavior and self-perceived stress may explain the conflicting results. Other potential explanations include ethnic differences and a small sample size in the study by Kim *et al*. However, taken together, these results indicate a differential influence of psychological stress on ischemic stroke subtypes, and emphasize the importance of considering subtypes in future prospective studies of psychological stress in stroke.

No association between self-perceived stress and functional outcome after 3 months was found. However, because patients with severe deficits in the acute stage were unable to give information on perception of psychological stress (n = 13), a selection type of bias underestimating a possible association between psychological stress and short-term outcome cannot be excluded.

Consistent with previous prospective studies using the same measure for self-perceived stress, we found an association for permanent but not periodic stress [2,11]. In line with this, it was recently reported from a large prospective study that ability to adapt to social adversity, rather than social adversity itself, was associated with stroke incidence [26]. Faster adaptation to adverse event exposure, measured as a strong sense of coherence, was associated with a reduced incidence of stroke. It can be speculated that a reduced capacity to for adaptation to social adversity may increase the susceptibility for perceived permanent stress. Moreover, as permanent stress may induce other biological responses as compared to periodic stress [27], the non-graded association is biologically plausible.

In the present study, we used a single-item questionnaire in which stress was described in psychological terms, such as feeling tense, irritable, anxious, or as having sleeping difficulties. It may be argued that this measure is too crude. However, one advantage of this measurement is that it has been shown to predict overall stroke as well as myocardial infarction in prospective studies [2,11]. It has also been shown to correlate with other constructs representing psychological stress including financial stress, stressful life events and locus of control [6]. Taken together, these results indicate a validity of the single-item question in investigations of cardiovascular risk.

However, the measure also has some obvious limitations. Each participant may interpret the questionnaire as a consequence of their own experience, and some may have confirmed the presence of psychological symptoms, such as feeling tense, irritable, anxious, or as having sleeping difficulties rather than stress. Therefore, the association between our measure of self-perceived psychological stress and ischemic stroke may be confounded by other forms of psychological distress and emotional reactions, including depression [28,29], anxiety or sleep difficulties. Moreover, as the questionnaire is a combined measure of exposure, ability to cope with, and report psychological stress, it does not give insight into subcomponents of psychological stress.

Because of the case-control design and retrospective assessment of stress, the association between psychological stress and ischemic stroke should be interpreted with caution. Patients who might believe their condition to be stress-induced may have introduced a recall bias. In addition, controls who refused to take part in the study may have had higher stress levels, potentially inflating differences between cases and controls. Consequently, it is inevitable that with the present design the association between self-perceived stress and stroke may be overestimated. Indeed, the association was stronger than that earlier reported from a prospective study using the same measure [11]. By contrast, exclusion of patients with large left hemisphere lesions due to aphasia and inability to answer to the questionnaire may potentially have introduced a bias towards an underestimation of the association, particularly since patients with large lesions in the right hemisphere and potential anosognosia were not excluded. However, the number of patients excluded due to aphasia was small (n = 8), and a major influence on our result from this kind of bias is therefore less likely. Selection and recall bias are also less likely to explain differences between subtypes.

The mechanisms by which permanent psychological stress may influence stroke risk are complex. Stress may be related to behaviors associated with an increased susceptibility for stroke, such as smoking, physical inactivity, and socioeconomic status [19]. Moreover, frequent or persistent activation of the sympathetic nervous system and the hypothalamic-pituitary-adrenal axis may also lead to hypertension and/or metabolic disturbances [27,30]. Indeed, in the present study, subjects reporting permanent self-perceived psychological stress displayed a more adverse risk factor profile as compared with subjects reporting no or periodic stress. However, the association between permanent self-perceived psychological stress and overall ischemic stroke, LVD, SVD, and cryptogenic stroke persisted after adjustment for these possible confounders, suggesting that there may also be a link independent of traditional vascular risk factors. These pathways may include direct effects of psychological stress on endothelial function [31], inflammation [32], atherosclerosis [33], and hemostasis [34].

The multivariate OR for ischemic stroke in subjects experiencing permanent psychological stress was as high as threefold. However, apart from the influence of recall bias and selection of controls mentioned earlier, the association may also be confounded by some other factor not controlled for in the multivariable model. Furthermore, cognitive impairment and previous cerebrovascular disease may influence coping abilities and perception of stress. As patients were included regardless of previous coronary and cerebrovascular events, we performed an analysis restricted to cases without previous clinical atherothrombotic disease. In this analysis the independent association between self-perceived psychological stress and ischemic stroke remained unchanged. Therefore, we believe it unlikely that inclusion of patients with previous stroke or cardiovascular diseases have had any major influence on our results. However, we cannot control for the potential confounding effect of preceding silent cerebrovascular disease without apparent clinical manifestations.

## Conclusion

Results from the present case-control study suggest an independent association between permanent self-perceived stress and ischemic stroke. A novel finding was that this association differed by ischemic stroke subtype. While the association was present in LVD, SVD, and cryptogenic stroke, it was not observed for CE stroke. However, because of the case-control design and retrospective assessment of stress, our observations should be interpreted with caution. Further prospective studies addressing the potential role for psychological stress as a risk factor for ischemic stroke are needed. In such studies ischemic stroke subtypes should be taken into consideration. Furthermore, to give insight as to which subcomponents of perceived stress that are of importance, more elaborate measures of psychological stress are suggested.

## Competing interests

The authors declare that they have no competing interests.

## Authors' contributions

KJ participated in design of the study and was responsible for recruitment and phenotyping of cases and controls, performed the statistical analysis and drafted the manuscript. PR participated in recruitment of patients and controls, and contributed in statistical analysis and drafting of the manuscript. AR participated in design of the study, recruitment of controls and statistical analysis. CB and CJ headed the study, participated in design of the study, and recruitment and phenotyping of cases. CJ also helped to draft the manuscript. All authors read and approved the final manuscript.

## Pre-publication history

The pre-publication history for this paper can be accessed here:



## References

[B1] Rozanski A, Blumenthal JA, Kaplan J (1999). Impact of psychological factors on the pathogenesis of cardiovascular disease and implications for therapy. Circulation.

[B2] Rosengren A, Tibblin G, Wilhelmsen L (1991). Self-perceived psychological stress and incidence of coronary artery disease in middle-aged men. Am J Cardiol.

[B3] Hemingway H, Marmot M (1999). Psychosocial factors in the etiology and prognosis of coronary heart disease: systemic review of prospective cohort studies. BMJ.

[B4] Iso H, Date C, Yamamoto A, Toyoshima H, Tanabe N, Kikuchi S, Kondo T, Watanabe Y, Wada Y, Ishibashi T, Suzuki H, Koizumi A, Inaba Y, Tamakoshi A, Ohno Y (2002). Perceived mental stress and mortality from cardiovascular disease among Japanese men and women: the Japan Collaborative Cohort Study for Evaluation of Cancer Risk Sponsored by Monbusho (JACC Study). Circulation.

[B5] Kario K, McEwen BS, Pickering TG (2003). Disasters and the heart: a review of the effects of earthquake-induced stress on cardiovascular disease. Hypertens Res.

[B6] Rosengren A, Hawken S, Ounpuu S, Sliwa K, Zubaid M, Almahmeed WA, Blackett KN, Sitthi-amorn C, Sato H, Yusuf S, INTERHEART investigators (2004). Association of psychosocial risk factors with risk of acute myocardial infarction in 11119 cases and 13648 controls from 52 countries (the INTERHEART study): case-control study. Lancet.

[B7] Moller J, Theorell T, de Faire U, Ahlbom A, Hallquist J (2005). Work related stressful life events and the risk of myocardial infarction. Case-control and case-cross over analyses within the Stockholm heart epidemiology programme (SHEEP). J Epidemiol Comm Health.

[B8] Orth-Gomér K, Leineweber C (2005). Multiple stressors and coronary disease in women. The Stockholm female Coronary Risk Study. Biol Psychol.

[B9] Pancioli AM, Broderick J, Kothari R, Brott T, Tuchfarber A, Miller R, Khoury J, Jauch E (1998). Public perception of stroke warning signs and knowledge of potential risk factors. JAMA.

[B10] Sug Yoon S, Heller RF, Levi C, Wiggers J, Fitzgerald PE (2001). Knowledge of stroke risk factors, warning symptoms, and treatment among an Australian urban population. Stroke.

[B11] Harmsen P, Rosengren A, Tsipogianni A, Wilhelmsen L (1990). Risk factors for stroke in middle-aged men in Göteborg, Sweden. Stroke.

[B12] House A, Dennis M, Mogridge L, Hawton K, Warlow C (1990). Life events and difficulties preceding stroke. J Neurol Neurosurg Psychiatr.

[B13] Andre-Petersson L, Hagberg B, Janzon L, Steen G (2003). Adaptive behavior in stressful situations in relation to postinfarction mortality results from prospective cohort study "Men Born in 1914" in Malmo, Sweden. Int J Behav Med.

[B14] Engstrom G, Khan FA, Zia E, Jerntorp I, Pessah-Rasmussen H, Norrving B, Janzon L (2004). Marital dissolution is followed by an increased incidence of stroke. Cerebrovasc Dis.

[B15] Gallo WT, Bradley EH, Falba TA, Dubin JA, Cramer LD, Bogardus ST, Kasl SV (2004). Involuntary job loss as a risk factor for subsequent myocardial infarction and stroke: findings from the Health and Retirement Survey. Am J Ind Med.

[B16] Kleinman Y, Korn-Lubetzki I, Eliashiv S, Abramsky O, Eliakim M (1992). High frequency of hemorrhagic strokes in Jerusalem during the Persian Gulf War. Neurology.

[B17] Macko RF, Ameriso SF, Barndt R, Clough W, Weiner JM, Fisher M (1996). Precipitants of brain infarction. Roles of preceding infection/inflammation and recent psychological stress. Stroke.

[B18] May M, McCarron P, Stansfeld S, Ben-Shlomo Y, Gallacher J, Yarnell J, Davey Smith G, Elwood P, Ebrahim S (2002). Does psychological distress predict the risk of ischemic stroke and transient ischemic attack? The Caerphilly Study. Stroke.

[B19] Truelsen T, Nielsen N, Boysen G, Gronbaek M, Copenhagen City Heart Study (2003). Self-reported stress and risk of stroke the Copenhagen City Heart Study. Stroke.

[B20] Öhlin B, Nilsson PM, Nilsson JA, Berglund G (2004). Chronic psychosocial stress predicts long-term cardiovascular morbidity and mortality in middle-aged men. Eur Heart J.

[B21] Kim JS, Yoon SS, Lee SI, Yoo HJ, Kim CY, Choi-Kwon S, Lee BC (1998). Type A behavior and stroke: high tenseness dimension may be a risk factor for cerebral infarction. Eur Neurol.

[B22] Jood K, Ladenvall C, Rosengren A, Blomstrand C, Jern C (2005). Family history in ischemic stroke before 70 years of age. The Sahlgrenska Academy Study on Ischemic Stroke (SAHLSIS). Stroke.

[B23] Samuelsson H, Hjelmquist E, Naver H, Blomstrand C (1996). Visuospatial neglect and an ipsilesional bias during the start of performance in conventional tests of neglect. Clin Neuropsychol.

[B24] Samuelsson H, Hjelmquist EK, Jensen C, Blomstrand C (2002). Search pattern in a verbally reported visual scanning test in patients showing spatial neglect. J Int Neuropsychol Soc.

[B25] Adams HO, Bendixen BH, Kappelle LJ, Biller J, Love BB, Gordon DL, Marsh EE (1993). Classification of subtype of acute ischemic stroke. Definitions for use in a multicenter clinical trial. Stroke.

[B26] Surtees PG, Wainwright NW, Luben RL, Wareham NJ, Bingham SA, Khaw KT (2007). Adaptation to social adversity is associated with stroke incidence: evidence from the EPIC-Norfolk prospective cohort study. Stroke.

[B27] McEwen BS (1998). Stress, adaptation, and disease. Allostasis and allostatic load. Ann N Y Acad Sci.

[B28] Salaycik KJ, Kelly-Hayes M, Beiser A, Nguyen AH, Brady SM, Kase CS, Wolf PA (2007). Depressive symptoms and risk of stroke: the Framingham Study. Stroke.

[B29] Surtees PG, Wainwright NW, Luben RN, Wareham NJ, Bingham SA, Khaw KT (2008). Psychological distress, major depressive disorder, and risk of stroke. Neurology.

[B30] Rosmond R (2005). Role of stress in the pathogenesis of the metabolic syndrome. Psychoneuroendocrinology.

[B31] Ghiadoni L, Donald AE, Cropley M, Mullen MJ, Oakley G, Taylor M, O'Connor G, Betteridge J, Klein N, Steptoe A, Deanfield JE (2000). Mental stress induces transient endothelial dysfunction in humans. Circulation.

[B32] Black PH (2003). The inflammatory response is an integral part of the stress response: Implications for atherosclerosis, insulin resistance, type II diabetes and metabolic syndrome X. Brain Behav Immun.

[B33] Wolff B, Grabe HJ, Volzke H, Lüdemann J, Kessler C, Dahm JB, Freyberger HJ, John U, Felix SB (2005). Relation between psychological strain and carotid atherosclerosis in a general population. Heart.

[B34] von Kanel R, Mills PJ, Fainman C, Dimsdale JE (2001). Effects of psychological stress and psychiatric disorders on blood coagulation and fibrinolysis: a biobehavioral pathway to coronary artery disease?. Psychosom Med.

